# Where Should the Surgical Neonates be Nursed?

**Published:** 2012-04-01

**Authors:** Siddarth Ramji, Neelam Kler, Avneet Kaur

**Affiliations:** 1 Director-Professor (Pediatrics), Department of Neonatology, MAMC, New Delhi, India; 2 Director and Head, Department of Neonatology, Sir Ganga Ram Hospital, New Delhi, India; 3 Consultant Neonatologist, Max Hospital Gurgaon, India

**Siddarth Ramji’s View-Point**

Surgical neonates need specialist medical and nursing care if their outcomes are to be improved. It needs a team of pediatric surgeons, neonatologists, pediatric anesthetists, and trained nurses. However, a key question in the management of these neonates is the place where they should be managed. A recent survey of neonatal surgical units in the UK in 2008 [1] revealed that surgical neonates were managed in a variety of wards – neonatal medical ICUs, Pediatric intensive care units, stand alone neonatal surgical units and pediatric medical/surgical wards. In most centers in the survey there was a flexible use of these wards and in most pediatric centers 2-3 flexible locations were used for the pre- and post-operative care of surgical neonates. The survey revealed that there were only 8 stand alone neonatal surgical units of the 64 pediatric surgical facilities surveyed. This scenario would be akin to the situation existing in most large hospitals in India which cater to neonatal surgical patients. While many may consider this an efficient way of utilizing available resources, it significantly dilutes the medical and nursing care provided to these neonates due to lack of specialized training by these personnel. Given the rarity of many of these surgical conditions, location of these neonates in range of wards will dilute the training of medical and nursing personnel in the care of these babies. In fact a report on the surgical care of neonates published by the British association of pediatric surgeons and the Royal College of Surgeons strongly recommended that surgical neonates should be concentrated in specialist pediatric surgical units which are adequately staffed with trained pediatric surgeons, medical and nursing personnel and adequate equipment to care for these infants. In the case of preterm infants and those with complex congenital malformations it recommended a multi-disciplinary approach especially involving neonatologists and specialist in perinatal medicine/obstetricians [2].

One may ask since most of the pre-operative and post-operative care of surgical neonates are similar to the medical needs of sick neonate, why not manage them with medical neonatal ICUs with support from the surgeons? It is true that many of the physiological needs such as temperature stability, fluid and electrolyte needs, ventilator support and TPN are similar to that of most medical neonatal ICUs [3], there are several reasons why this may not be the best location to care for these neonates. Firstly most medical NICUs in India are already over burdened with sick neonates and are not large enough to dedicate beds for surgical neonates. Secondly, since these surgical problems are relatively uncommon amongst all births with their specialized needs, the medical and nursing staffs in medical NICUs have insufficient training and exposure to understand the special needs of surgical neonates which could affect their care. Thirdly, concentrating surgical neonates in non-surgical neonates would dilute the training and experience of specialist pediatric surgeons in managing these infants; an objective that would be better achieved if they were concentrated in pediatric surgical units. Most pediatric surgeons (as also revealed by the BAPS survey) [1] are not actively involved in ventilating surgical neonates. However, it would be easier to have one staff or a link with a neonatologist and train the nursing staff in the surgical unit in the care of preterm or ventilated surgical neonates. This would also over time increase the skills of the pediatric surgeon in ventilating these newborns. Concentrating surgical neonates within surgical units would also have the advantage of easy access to the closely located operating room, minimizing transportation within large hospitals.

**Neelam Kler and Avneet Kaur’s View-Point**

Advancements in medical science over the last two decades have resulted in development of specialized fields like neonatology, paediatric/neonatal surgery, paediatric anaesthesia and neonatal nursing. This in turn has resulted in improved outcomes for surgical neonates. Gone is the era when adult general surgeons would operate on children but did not have the expertise in treating newborn surgical problems. Nowadays, in the developed world, multidisciplinary approach has evolved where pediatric/neonatal surgeons work in tandem with the perinatologists, obstetricians, neonatologists, pediatric cardiologists, pediatric neurosurgeons, pediatric anesthesiologists to optimize the management and outcome in these patients.

Neonatal surgery encompasses not only the surgical details and techniques of correction of the congenital malformations, but also understanding of the neonatal physiology, the patho-physiology, the spectrum of anomalies, genetics, the diagnostic modalities and advances in neonatal intensive care. In no other components of pediatric surgery, the pre-operative preparation and post-operative management is as critical for a successful outcome as in neonatal surgery [4].

This is particularly important in the case of preterm and term low birth weight babies, where temperature regulation, fluid balance, assessment of metabolic disturbances, ventilation, management of nutrition etc are vital for a successful outcome. Such patients are best managed under a neonatologist who are trained and have expertise in managing the neonate as a whole. Pediatric/neonatal surgeons usually have a myopic vision which is focused on the surgical issue at hand. These patients thus should ideally be taken care of in a neonatal intensive care unit. The era when surgical neonates used to be housed in one of the many cubicles at the end of children’s surgical ward is a thing of the past.

A survey of neonatal surgical units in the UK in 2008 [1] revealed that surgical neonates were managed in a variety of wards – NICUs, PICUs and stand alone neonatal surgical units. In the 23 centres surveyed there were 64 different ward areas used. Given the very specialist nature of neonatal surgery and in particular the pre and post-operative care involved, it is concerning to see that there are so many different ward areas that may be used for the admission of surgical neonates. It is difficult to maintain high levels of medical and nursing experience and expertise in so many different ward areas. Conversely, having access to a number of wards with beds available on a flexible basis should help with bed availability.

The situation in a developing country like India which has an acute shortage of trained specialists and staff is very different. There are few centres providing neonatal surgery, considerable variation in the volume of care and an acute shortage of paediatric/neonatal surgeons. The limited surgeons results in time constraints and inability to devote sufficient time to medical care. It is difficult to envisage specialized and dedicated neonatal surgical units being managed exclusively by surgeons (this may be a possibility only in a large centre with sufficient case volume and staffing). A more practical scenario for expansion of neonatal surgery in vast country like India will be to have these patients managed in Nursery/NICUs with active involvement of paediatric surgeon in post operative care (older paediatric surgical population can be managed in PICUs/child surgical wards). This is the model which is presently being followed at most centres in India.

Few would argue that the most appropriate specialist to manage a very low-birth-weight preterm baby will be a neonatologist. The neonatologist is also the best person to manage a term neonate whose needs are very different from a small child. Neonatal surgical care modules and curricula have been developed, both for the trainee doctors as well as nurses to improve their expertise in care of surgical neonate [5]. New concepts like operating the surgical neonates in NICU itself have emerged [6,7]. Therefore the best place to manage surgical neonates is a neonatal intensive care unit (NICU) with active involvement of paediatric surgeons.

## Footnotes

**Source of Support:** Nil

**Conflict of Interest:** None declared

**Editorial Comments**

Though it is recommended that all the teaching pediatric surgery departments should have independent surgical neonatal intensive care unit (NICU), only very few departments of pediatric surgery in some premiere institutes such as AIIMS New Delhi, PGIMER Chandigarh have independent surgical NICU with the desired infrastructure and personnel. The oft-quoted reasons for not having such surgical NICU include not having adequate numbers of pediatric surgical residents, lack of resources and infrastructure, inadequate exposure to the advanced gadgetries including ventilators etc. The spectrum of anomalies and diseases is so vast that it is difficult to spare time and do justice to the running of such specialized unit. However, neonatal surgery is the flagship component of pediatric surgery and we should all strive to develop and maintain specialized surgical NICUs. In few countries such as Brazil and United Kingdom, there is a provision of an additional year of training in neonatal surgery after the designated years of pediatric surgery training. The department of pediatric surgery in SMS Medical College, Jaipur has a faculty post earmarked for neonatal surgery. Such provisions will go in a long way to the advancement of neonatal surgery in this part of the world.

The other extreme of arrangement is occasionally practiced in few private corporate hospitals in metro cities of India where a neonatologist is available in the operation theatre where neonate is being operated. This appears overkill to my mind. To conclude, till we reach a stage where we could develop and maintain surgical NICUs, we should rotate the pediatric surgical residents to neonatology units for a period of 2-4 weeks during their training so as to sensitize them and give them enough exposure to the basics of neonatal intensive care.

